# The Effect of Thermally Robust Ballistic Mechanisms on Climatic Niche in Salamanders

**DOI:** 10.1093/iob/obac020

**Published:** 2022-08-13

**Authors:** Sarah T Friedman, Martha M Muñoz

**Affiliations:** Department of Ecology and Evolutionary Biology, Yale University, New Haven, CT 06511,USA; Department of Ecology and Evolutionary Biology, Yale University, New Haven, CT 06511,USA

## Abstract

Many organismal functions are temperature-dependent due to the contractile properties of muscle. Spring-based mechanisms offer a thermally robust alternative to temperature-sensitive muscular movements and may correspondingly expand a species’ climatic niche by partially decoupling the relationship between temperature and performance. Using the ballistic tongues of salamanders as a case study, we explore whether the thermal robustness of elastic feeding mechanisms increases climatic niche breadth, expands geographic range size, and alters the dynamics of niche evolution. Combining phylogenetic comparative methods with global climate data, we find that the feeding mechanism imparts no discernable signal on either climatic niche properties or the evolutionary dynamics of most climatic niche parameters. Although biomechanical innovation in feeding influences many features of whole-organism performance, it does not appear to drive macro-climatic niche evolution in salamanders. We recommend that future work incorporate micro-scale environmental data to better capture the conditions that salamanders experience, and we discuss a few outstanding questions in this regard. Overall, this study lays the groundwork for an investigation into the evolutionary relationships between climatic niche and biomechanical traits in ectotherms.

## Introduction

From escaping predators to locating mates and acquiring food, many crucial organismal functions in ectotherms are thermally dependent (Levins 1968; Huey and Stevenson 1979). All these activities result from the coordinated movement of underlying musculature, and the contractile properties of muscle are highly sensitive to temperature changes (Bennett 1984, 1985). Therefore, the performance of any muscular movement and the essential activities that rely on such movement are directly related to temperature (James 2013). Beyond the thermal boundary, proteins denature, physiological processes decline, and organismal function rapidly deteriorates (Angilletta 2009). Biomechanical innovations have arisen that relax the temperature dependence of certain muscular-based mechanisms. Latch-mediated elastic movements, like the quick-closing maws of trap-jaw ants and bullet-fast strikes of mantis shrimp, have evolved numerous times across many distantly related organisms (Patek et al. 2004, 2006; Ilton et al. 2018; Longo et al. 2019). These biomechanical systems function by deforming elastic material to store energy; following the switch of a latch, this energy can then be released in a single, rapid movement (Patek et al. 2011). Compared to purely muscle-based motion, spring-based mechanisms can drastically increase the power, acceleration, and functional range of movements (Patek et al. 2011; Roberts and Azizi 2011). Critically, such movements are thermally robust: Elastic-based mechanisms operate equally well (or better) across a broader range of temperatures without substantial performance decrement (Deban and Lappin 2011; Quenta Herrera et al. 2018; Deban and Anderson 2021).

The ecological and evolutionary consequences of elastic mechanisms are potentially multi-fold. Given that climatic features (e.g., temperature and moisture) are major determinants of species’ distributions (Pigot et al. 2010; Searcy and Shaffer 2016; Baken et al. 2021), possessing a trait that grants the ability to suitably operate across a broader range of temperatures could afford organisms greater niche breadth (e.g., by expanding the range of suitable climatic conditions), extension into novel niche space (e.g., via access into new climatic regions), and/or increase the number of suitable microhabitats (e.g., unlock novel resources). There are multiple instances in the literature of traits, particularly those with direct physiological consequences, altering climatic niches. For instance, higher surface area to volume ratio of different salamander species is associated with a shift toward warmer and wetter climates (Baken et al. 2020), an increase in body size and locomotor performance in Tibetan toads promotes climatic niche expansion (Lin et al. 2021), and woody plants occupy smaller climatic spaces than herbaceous lineages (Smith and Beaulieu 2009). Any mechanism of increasing niche space can also result in enlarged geographic ranges (Buckley and Kingsolver 2012) by facilitating the occupation of a wider range of habitats and environmental conditions (Slatyer et al. 2013). Thermal specialization, in particular, is associated with narrower geographic ranges and increased extinction susceptibility in salamanders (Markle and Kozak 2018). Whereas many authors have coalesced around the idea that elastic mechanisms may grant ecological benefits (Anderson et al. 2014; Deban et al. 2020; Olberding and Deban 2021), empirical tests of these hypotheses are lacking, particularly at a macroevolutionary scale.

Extending these ideas, traits that expand or alter the climatic niche that a species occupies can also influence the dynamics of climatic niche evolution. Yet, the relationship between rates of niche evolution and niche breadth is complex and contentious (Fisher-Reid et al. 2012; Sexton et al. 2017). Traits that relax the conditions that a species can tolerate may confer increased niche lability because species with a wider climatic tolerance will also tend to have larger ranges, with more potential for vicariant speciation through evolutionary time. Furthermore, more specialized species may be evolutionarily constrained due to phenotypic trade-offs (Futuyma and Moreno 1988). Alternatively, there is an argument that narrower niches may promote adaptive diversification, as specialization can drive finer partitioning of niche space and population subdivision (Kassen 2002; Dyer et al. 2007). We also note that traits themselves can also alter niche evolutionary dynamics: Planktivorous damselfishes have faster rates of niche evolution than herbivorous species (Litsios et al. 2012), and salamanders with longer larval periods experience reduced rates of niche evolution (Weaver et al. 2020). Thus, there is theoretical potential for a link between thermally robust elastic mechanisms and rates of niche evolution, particularly via expansion of niche breadth. Evaluating such questions in the context of elastic mechanisms for niche evolution remains an open, and potentially insightful, area of research.

Salamanders present an ideal system in which to test hypotheses about the relationship between biomechanical mechanisms and climatic niche evolution. With over 700 species, salamanders have diversified extensively across the Northern Hemisphere into northern South America, and occupy a correspondingly wide range of climatic regimes, microhabitats, and feeding ecologies (Wake 2009). Salamanders employ a variety of prey capture modes, which vary somewhat predictably with habitat (Herrel et al. 2019). For example, aquatic species generally rely on suction feeding and/or jaw prehension, whereas terrestrial species tend to use tongue protrusion. Tongue protrusion is biomechanically variable in salamanders, with some species relying on purely muscle-based protrusion and others using a modified tongue structure to facilitate spring-based, high-speed prey capture (Lombard and Wake 1976; Deban et al. 2020; Scales et al. 2020). In species with purely muscle-based tongue protrusion, tongue attachment is wide, with a shorter tongue skeleton, and a large tongue pad that only protrudes a relatively short distance from the mouth (Deban et al. 2020). By contrast, species with elastic tongue projection lack myofiber attachments and possess elongated connective tissue. During feeding, the tongue skeleton separates from the tongue projector muscles, which has resulted in dramatically altered kinematic features, like increased displacement distance (Scales et al. 2016). For example, studies on the terrestrial salamander, *Plethodon metcalfi*, which solely rely on muscle, demonstrated a maximum tongue protrusion velocity of 1.73 s over 40% of its body length (Deban and Scales 2016). By contrast, spring-based tongue projection in *Hydromantes supramontis* can reach a distance of up to 80% of body length in under 10 ms (Deban et al. 1997). In spring-based systems, energy is stored in collagenous aponeuroses prior to movement, decoupling muscle activation from tongue protrusion. As a result, elastic-based tongue projection can operate across a wide range of temperatures without substantial performance decrement (Deban and Lappin 2011; Deban and Richardson 2011; Anderson et al. 2014; Deban et al. 2020). Ballistic feeding, therefore, has the potential to relax thermal constraints on feeding performance, and may be an engine for climatic niche diversification.

Using salamanders as a case study, we explore some of the implications of biomechanical innovations for niche evolution and geographic range size. We leverage the repeated evolution of a ballistic tongue with a phylogenetic framework to test whether the presence of an elastic mechanism (1) enhances access to colder environments and, correspondingly, expands macro-climatic niche breadth and geographic range size; (2) is associated with specific microhabitat usage; and (3) alters climatic niche evolution. We discuss the role that biomechanical innovation plays in shaping diversification dynamics, building on a growing body of literature that integrates ecology, evolution, and whole-animal physiology.

## Methods

### Data collection

As the prey capture mechanism tends to be conserved at the genus level (Deban et al. 2020), a broad phylogenetic dataset is necessary to investigate climatic patterns associated with feeding evolution in salamanders. We compiled an extensive dataset using primary literature, IUCN (IUCN 2021), and AmphibiaWeb (AmphibiaWeb 2021), which encompasses all species of salamanders in which the mechanism of tongue protrusion is formally described (Supplementary data, Table S1). This dataset contains 78 salamander species across 25 genera and 4 families and spans many of the geographic regions that salamanders occupy, as well as much of the ecological diversity (habitat, diet, etc.) found in the clade. Although we note that some species can use both tongue protrusion and suction feeding as well as jaw prehension to acquire prey depending on context and life stage (Herrel et al. 2019), we only included species that rely primarily on tongue protrusion as adults. As microhabitat use may influence exposure to ambient climatic conditions, we also incorporated previously published fine-scale microhabitat categories for each species into analyses (Fabre et al. 2020). Based on their adult microhabitat preferences, species were classified using one of the following categories: semi-fossorial, aquatic, semi-aquatic, terrestrial, arboreal, aquatic species living in caves, and terrestrial species living in caves.

Geographic occurrence information for each species was downloaded from the Global Biodiversity Information Facility (GBIF; www.gbif.org) using the R package dismo (Hijmans et al. 2011). These data were then filtered to remove outliers, erroneous data points, and spatially autocorrelated points using the R package CoordinateCleaner (Zizka et al. 2019), resulting in a total of 199,104 observations (mean number of observations per species: 2,804) with considerable variance in the number of observations across species. We were unable to estimate ranges for the two species with too few observations remaining after the cleaning procedure. We estimated the range size (km^2^) for each species as the 95% minimum convex polygon fitted around the verified occurrence points. This range estimation method avoids introducing bias from geographically extreme points (Burgman and Fox 2003), as well as the pitfalls of more complex species distributional models (Araújo and Guisan 2006; Warren et al. 2020), and is roughly consistent with reported ranges for these species (AmphibiaWeb 2021). To incorporate phylogenetic relationships into our analyses, we used a time-calibrated phylogeny (Bonett and Blair 2017) pruned to the species for which both feeding mode and habitat data were available, leaving 71 species with complete data for further analyses.

For each verified occurrence point, we extracted altitude and 19 bioclimatic variables from the WorldClim database (www.worldclim.org) at a 2.5-arc min resolution (∼4.5 km at the equator). These variables represent standard annual measures of temperature and precipitation (Hijmans et al. 2005) and are commonly used in studies of niche evolution in amphibians (Quintero and Wiens 2013; Bonetti and Wiens 2014). Although our predictions for this study relate specifically to altered temperature regimes, precipitation and temperature are generally tightly coupled and the temperature–moisture combination has been shown to strongly predict salamander distributions (Fisher-Reid et al. 2012; Peterman and Semlitsch 2014; Riddell et al. 2017; Farallo et al. 2020). Thus, we explored environmental variables related to moisture to systematically evaluate changes in salamander climatic niche associated with feeding mode. To account for multicollinearity, we estimated Pearson correlations between all 19 bioclimatic variables and only retained variables that had a correlation coefficient of less than 0.7 (Dormann et al. 2013). Therefore, our dataset consisted of the median altitude and five bioclimatic variables for each species (Supplementary data, Fig. S1): mean diurnal temperature range (BIO2), maximum temperature of the warmest month (BIO5), minimum temperature of the coldest month (BIO6), precipitation of the wettest month (BIO13), and precipitation of the driest month (BIO14).

### Statistical analysis

We used a phylogenetic comparative method approach to evaluate the evolutionary relationship between feeding mode and microhabitat, range size, and climatic variables across species. For each species, we calculated the median, maximum, and minimum values, as well as the standard deviation for each climatic variable. As the climatic variables are on different scales (e.g., temperature measured in °C, precipitation measured in mm, etc.), we first normalized the values to allow for multivariate analyses (Adams and Collyer 2019). We then performed a series of phylogenetic regressions (phylogenetic generalized least-squares [PGLS]) using the R package geomorph (Adams et al. 2019) to examine the relationship between feeding mode and each of the climatic variables, as well as altitude and range size. These regressions allowed us to determine if ballistic and muscular-based feeding modes were associated with divergence in any of the individual abiotic variables. We assessed significance via a randomized residual permutation procedure in the R package RRPP (Collyer and Adams 2018) and used a Bonferroni correction to account for multiple comparisons. Additionally, we used a phylogenetic MANOVA with microhabitat as a covariate for a more composite analysis to evaluate if abiotic conditions differ with feeding mechanism. Lastly, we used a linear discriminant analysis implemented in the MASS R package (Venables and Ripley 2002) to determine which climatic features best distinguish elastic and non-elastic species. As the evidence for ballistic feeding in *Chioglossa lusitanica*, in particular, is somewhat indirect (Stinson and Deban 2017) and because this species would represent an independent evolutionary origin of ballistic feeding, we repeated all analyses with this species removed to ensure it does not bias our findings.

### Evolution of feeding mode

We first estimated the phylogenetic signal associated with elastic and non-elastic feeding to assess the statistical dependence of salamander feeding mode on relatedness. Using the geomorph R package (Adams et al. 2019), we implemented a multivariate generalization of Blomberg's K with 1000 iterations to assess significance. Next, to determine if elastic-based feeding modes were associated with faster rates of climatic niche evolution, we first generated 100 stochastic character maps (simmaps) of feeding mode (ballistic versus muscle-based mechanism) under the all-rates-different model (Bollback 2006; Revell 2012). We determined the best-fit model by comparing log-likelihoods of the *Q* matrices from models that allowed for equal, symmetric, and asymmetric rate transitions between feeding modes. Using the R package “OUwie” (Beaulieu and O'Meara 2014), we then implemented a model-fitting framework on each environmental variable (climatic features, range size, and altitude) to compare rates of evolution between species that rely on different feeding mechanisms. To test whether rates of evolution vary between elastic and non-elastic species, we compared two models: (1) a single-rate Brownian motion (BM1) model, which does not allow for the rate parameter (σ^2^) to vary with feeding mode, and (2) a multi-rate Brownian motion (BMS) model, which fits a different rate parameter to each feeding mode under maximum likelihood. We also checked results of the OUwie analyses for positive eigenvalues, which indicate reliable parameter estimates. Model fit was evaluated using a modified Akaike information criterion that accounts for small sample sizes (AICc). To ensure we had sufficient statistical power to distinguish between evolutionary models, we simulated data under the BMS model and re-ran the model-fitting procedure over 100 simmaps. Recovering the initial model and parameters would indicate an ability to distinguish between the two BM models. All analyses were performed in R (R Core Team 2017).

## Results

Whereas salamander species that rely on ballistic tongue projection tend to occupy terrestrial (*n* = 17), aquatic (*n* = 9), and arboreal (*n* = 8) habitats, species that use muscular tongue protrusion are primarily terrestrial (*n* = 20) and aquatic (*n* = 11; Supplementary data, Fig. S2). These results are slightly surprising, as aquatic species tend to rely on suction feeding when submerged, which is thought to be functionally antagonistic to specialized tongue protrusion (Deban and Marks 2002). Variation in range size across salamanders spans approximately four orders of magnitude, from *Eurycea waterlooensis*, occupying roughly 17.3 km^2^ to *Salamandrella keyserlingii* at an estimated 1.3 × 10^7^ km^2^.

### Statistical analyses

**Fig. 1:**
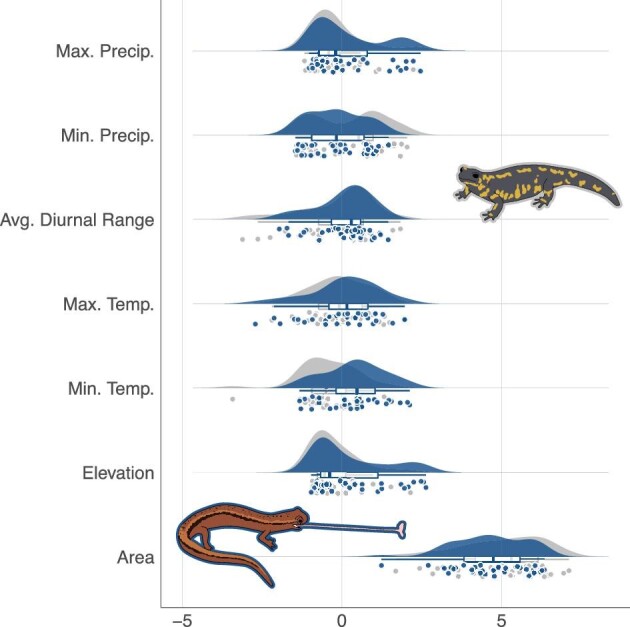
Climatic variable distributions do not differ between species with elastic and muscular mechanisms. Normalized values of each climatic variable across salamander species with elastic (blue) and non-elastic (gray) feeding mechanisms. Representative species from each feeding mode category are illustrated (blue outline: *Eurycea cirrigera*; gray outline: *Salamandra salamandra*).

We find that species utilizing ballistic feeding do not occupy larger ranges than species relying on purely muscle-based feeding. Range estimates for species with elastic- and muscular-based feeding mechanisms are broadly overlapping (Fig. 1) and are statistically indistinguishable after accounting for phylogeny (*P* > 0.05). Similarly, all the individual phylogenetic ANOVAs (across median, maximum, minimum, and standard deviation) comparing abiotic conditions for elastic and non-elastic species resulted in weak effect sizes and statistically indistinguishable values (Fig. 1, Supplementary data, Table S2). The phylogenetic MANOVA, which included microhabitat as a covariate and compared all abiotic variables together, likewise revealed no differences in climatic conditions among ballistic and muscular feeding mechanisms. Linear discriminant analysis, which determines the climatic variables that most strongly differentiate between ballistic and muscular feeders, revealed that range area is the key factor, though, combined with the ANOVA results, it is a weak differentiator (Supplementary data, Table S3). Removing *Chioglossia lusitanica* from the dataset does not alter any of our findings (Supplementary data, Table S4).

### Evolution of feeding mode

**Fig. 2:**
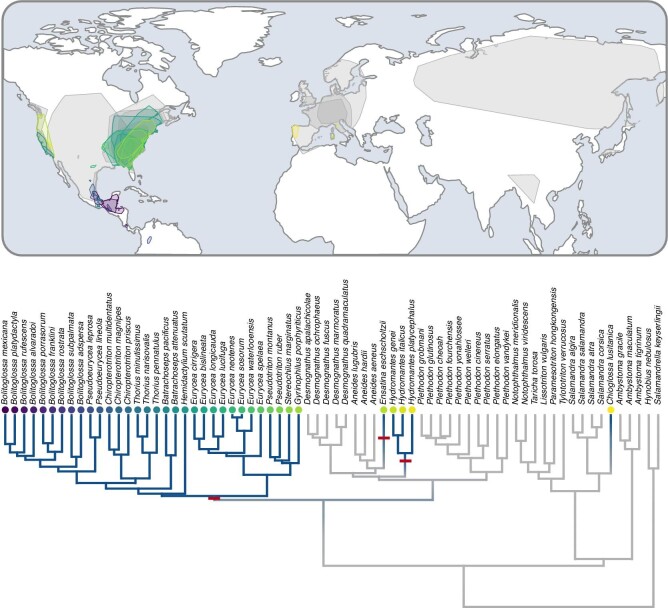
Ranges largely overlap between elastic and muscular species, despite elastic feeding having evolved at least three different times (denoted by the red bars). Range estimates for all species and a representative stochastic character map of feeding mode (blue: elastic mechanism; gray: non-elastic mechanism). Ballistic feeders in western North America, eastern North America, Central America, and Europe (species-specific ranges differentiated by the different colored points) share geographic space with non-ballistic feeders (gray).

Consistent with previous work, we find that elastic tongue protruding mechanisms have likely evolved at least three times across salamanders: (1) at the base of the clade, including *Eurycea* and *Bolitoglossa*, (2) in *Hydromantes*, and (3) in *Ensatina* (Fig. 2). There is potential for a fourth origin of ballistic feeding in *Chioglossa* based on the power and velocity of tongue protrusion demonstrated in this species (Stinson and Deban 2017), but a more detailed study of SAR muscle morphology is needed to confirm the underlying feeding mechanism. Furthermore, there is some evidence that ballistic mechanisms may have been lost once, at the base of the clade, including *Plethodon* + *Desmognathus.* Together, these findings imply that it is easier for salamanders to gain elastic mechanisms of tongue projection than to revert to the ancestral muscular-based tongue protrusion. Indeed, this may reflect the morphological lability of feeding musculature throughout the evolutionary history of salamanders (Scales et al. 2020). Phylogenetic signal is very high among ballistic feeders (*K* = 1.13, *P* = 0.001), particularly in contrast with muscular feeders (*K* = 0.41, *P* = 0.001). This finding implies that ballistic feeding is largely restricted to closely related species and that phylogeny has high explanatory power for the evolutionary history of feeding mechanisms across salamanders. The strong phylogenetic signal in the dataset is likely why we find no significant differences in any of the climatic variables between ballistic and muscle-based feeding modes.

Multi-rate Brownian motion is favored in three traits: altitude, minimum temperature of the coldest month (BIO6), and precipitation of the driest month (BIO14). However, given that AICc scores differ by very little between models (<2) for both altitude and BIO6, and in line with best practices of model fitting (Burnham and Anderson 2004), we consider BIO14 as the only trait with a detectable difference in rates between feeding mechanisms (Supplementary data, Table S5). Species with elastic-based tongue projection have marginally slower rates of minimum precipitation evolution when compared to species with muscle-based feeding modes (Supplementary data, Fig. S3). Meanwhile, single-rate BM is unambiguously favored for range area, maximum temperature of the warmest month (BIO5), and minimum temperature of the coldest month (BIO6), implying feeding mode is not associated with shifts in rates of evolution in these climatic variables (Supplementary data, Table S5). Our simulations showed that we have acceptable power to distinguish between evolutionary models, as all 100 recovered BMS as the best-fitting model for data simulated under BMS evolution (Supplementary data, Table S6).

## Discussion

Temperature constraints play a major role in many aspects of ectotherm ecology, physiology, and evolution, from placing limits on geographic ranges to restricting the activity patterns of essential biological processes (Angilletta et al. 2002; Angilletta 2009). Extending these concepts, researchers have speculated that temperature can also mediate the link between biomechanical processes and ecological interactions, like prey acquisition (Deban and Lappin 2011; Anderson et al. 2014; Deban et al. 2020), although macroevolutionary tests are lacking. Here, we hypothesize that the presence of a thermally robust elastic-based feeding mechanism would both expand the climactic niche and alter rates of climatic niche evolution in salamanders. Contrary to our expectations, however, we find no statistical evidence that species with ballistic tongue projection occupy expanded geographic ranges or regions with different climatic conditions from other salamander species, although they do tend toward more terrestrial and arboreal habitats when compared to muscle-based tongue protruders. Aside from minimum precipitation, which evolves faster in species with muscle-based tongue protrusion (a result we unpack in detail below), we find scant evidence that the biomechanical properties of feeding mechanisms influence climatic niche evolution. Together, our results imply that there is little potential for biomechanical features to influence the dynamics of climatic niche evolution. We speculate that there are several explanations for the lack of correlation between elastic mechanisms and climatic niche, which broadly fall into three domains: biomechanical, physiological, and ecological. Through their interpretation below, we argue that complex interactions among these features limit generalizable linkages between biomechanical innovation and climatic niche evolution.

### Mechanisms decoupling biomechanics and climatic niche

Our findings that elastic-based mechanisms are not associated with shifts in climatic niche evolution in salamanders may reflect constraints or selective biases at the biomechanical level. The performance boost afforded by elastic energy storage in the tongue apparatus is substantial and has clear evolutionary advantages beyond its thermal properties. In other words, the thermal robustness of elastic mechanisms may not be the primary target of selection. For example, *Bolitoglossa* is capable of amplifying the power of its tongue muscles 100 times (Deban et al. 2020), allowing tongue projection to occur in a fraction of the time needed by other species. The impressive power output and acceleration of the feeding apparatus are beneficial to feeding performance in salamanders, which, otherwise, rely on slow movements for locomotion and prey capture (Deban and Richardson 2011). Correspondingly, ballistic tongue feeding may result from dietary specialization that has little to do with climatic niche properties. We also note that, while tongue protrusion can have an elastic basis in some species, other feeding mechanisms, such as tongue retraction, are nonetheless thermally dependent (Deban and Richardson 2011; Anderson et al. 2014). Likewise, it is possible that the thermal dependence of other essential biomechanical systems, such as locomotion, may preclude climatic evolution or geographic shifts regardless of the tongue projection mechanism. Although we know that biomechanical shifts can influence both the pattern and rate of evolution (Holzman et al. 2012; Muñoz et al. 2017, 2018; Muñoz 2019), it is unclear in salamanders how the other mechanical demands, like locomotion, might impact climate niche evolution.

Salamanders may also be subjected to climatic niche constraints for reasons beyond the thermal dependence of biomechanical feeding systems. Many physiological processes can impose harsh limits to the environmental conditions an organism can endure. Salamanders are well known for their permeable skin, which acts as a respiratory surface but also provides little resistance to water loss, rendering them vulnerable to desiccation, particularly in lungless species (Spight 1968). Thus, precipitation and the overall moisture content of the environment are important predictors of salamander distributions (Fisher-Reid et al. 2012; Peterman and Semlitsch 2014; Riddell et al. 2017).

Likewise, temperature strongly covaries with precipitation, and heat stress is an acute selective force for terrestrial salamanders (Spotila 1972; Peterman and Semlitsch 2014). Studies have shown that salamanders are highly sensitive to vapor pressure deficit, which increases in warmer and drier conditions, and can shape the capacity of species to adapt along abiotic gradients such as those imposed by elevational ranges (Riddell and Sears 2015) and latitude (Clay and Gifford 2018). Combined, temperature and hygric conditions may impose unyielding boundaries to climatic niche evolution in salamanders, even in species with more thermally robust feeding systems. Salamander distributional patterns are strongly shaped by climatic conditions with little concurrent trophic differentiation (Kozak and Wiens 2006, 2010; Adams et al. 2009), indicating that whole-organism physiology may be a greater source of climatic constraint than feeding ecology in this system.

Despite the extensive physiological constraints placed on salamanders, it is still somewhat unexpected that we find no effect of niche expansion in species with an adaptation that theoretically affords it. Although there is evidence that macrohabitat can be a decent (albeit imperfect) predictor of micro-climatic niche (Farallo et al. 2020), and these climatic data have been used for macroevolutionary studies on salamanders (Baken et al. 2021), the lack of a significant effect may indicate we are not adequately characterizing the environmental conditions of salamander species. We note that salamanders are sensitive to micro-level perturbations in environmental conditions that are not entirely captured by broad-scale variables used here (Ficetola et al. 2018). Microhabitat use may obscure patterns in broad-scale climatic variables across salamanders. For example, many of the species included in these analyses spend a large portion of their time submerged in water, which can buffer air temperature fluctuations. Likewise, terrestrial species can exploit favorable microhabitats, like under leaf litter or within caves, that are buffered from broader regional climate, though we note that conditions can be dependent on surface conditions (Ficetola et al. 2018). Ultimately, feeding mode appears to have more deterministic power for microhabitat than it does for macro-climatic niche. Although incorporating fine-scale humidity and soil temperature would more accurately reflect the conditions experienced by these species, the existing databases, while promising for future work, are not yet extensive enough to encompass the phylogenetic and geographic scales of this study (Lembrechts et al. 2020; Baken et al. 2021).

Activity patterns may present another potential confounding variable to this study. Instead of exhibiting spatial shifts in climatic niche, salamanders with elastic feeding mechanisms may simply alter their habitat use and temporal activity patterns. Species with elastic-based mechanisms of tongue projection may be capable of foraging over longer periods of time without performance loss throughout daily temperature fluctuations (Olberding and Deban 2021). Unfortunately, empirical data on activity patterns across species of salamanders are currently lacking and are difficult to obtain due to the nocturnal and enigmatic nature of many salamander species, although we note that climatic conditions have appreciative predictive power for salamander surface activity (Peterman and Gade 2017; Farallo et al. 2020; Gade et al. 2020). The presence of an elastic-based mechanism may also allow individuals to rely less on behavioral-mediated temperature adjustments, which have been extensively documented in ectotherms (Bogert 1949; Farallo et al. 2018; Muñoz and Losos 2018; Burress and Muñoz 2022; Muñoz 2022). Though behavioral changes can allow organisms to evade thermal stress, this strategy is not without significant ecological costs in the form of time and energy (Huey 1974; Vickers et al. 2011). Any adaptation that reduces the need to behaviorally thermoregulate may offer considerable evolutionary advantages, such as allowing organisms to prioritize other physiological requirements (e.g., reducing evaporative water loss) above maintaining muscle temperature (Deban and Richardson 2011). Presently, the implications of elastic-based mechanisms on temporal niche and behavior are poorly understood and would benefit from further research.

### Biomechanics and climatic niche evolution

Although we recover very few differences in the rates of climatic evolution of species with ballistic and muscular tongue protrusion mechanisms, the one exception is minimum precipitation. Here, we find that species with a muscular-based feeding mechanism exhibit slightly faster niche lability in more arid environments. While this finding suggests that possessing a thermally robust feeding mechanism may constrain species along the precipitation gradient, it is interesting that we find no similar effect with maximum precipitation or concomitant differences in rates of temperature evolution. In light of the extensive overlap in climatic niche between ballistic and muscular tongue protruders, the mechanistic explanation for this pattern is unclear. Rather than a direct consequence of the feeding mechanism itself, we suspect the most likely driver of this finding is the covariation between latitude and feeding mode in this dataset. The majority of species with ballistic tongues in this dataset are found at lower latitudes in Mexico and Central America, regions with relatively high levels of precipitation and generally stable environmental conditions through time. Therefore, there is less opportunity to diversify into environments with very different abiotic conditions, limiting climatic niche evolution. By contrast, many of the species that rely on muscle-based tongue protrusion occupy temperate environments in North America and Europe, where species are exposed to much greater variation in environmental conditions, and, in particular, regions with less precipitation (Vázquez and Stevens 2004). Consistent with our interpretation, a latitudinal effect on rates of climatic niche evolution has been demonstrated in birds (Lawson and Weir 2014). Considering the lack of a mechanistic explanation for the minor rate difference in minimum precipitation and the absence of rate differences in any other climatic parameter, we conclude that feeding mode has limited capacity to affect the dynamics of niche evolution on a macroevolutionary scale.

## Conclusions

To our knowledge, this is the first study to investigate the role of elastic-based mechanisms in driving climatic niche evolution. Though we do not find an association between climate and feeding mechanism, this work informs our understanding of the potential ecological and evolutionary consequences of biomechanical features. Salamanders are unusually constrained by abiotic factors, and it remains unclear whether the lack of relationship found here is indicative of broad-scale patterns across ectotherms or whether these results are a consequence of our chosen study system. Thus, this study lays a foundation for exploration into the evolutionary relationships between climatic niche and biomechanical traits in ectotherms. Further work on this topic will undoubtedly be improved by fine-scale microhabitat, behavior, and climate data.

## Funding

Funding for this work was provided by the National Science Foundation (DEB-2039476) to M.M.M. and by a G. Evelyn Hutchinson Environmental Postdoctoral Fellowship to S.T.F.

## Conflict of interest

The authors declare no competing interests.

## Supplementary Material

obac020_Supplemental_File
