# Supplementary material for: The Effect of Thermally Robust Ballistic Mechanisms on Climatic Niche in Salamanders
Source: Integr Org Biol. 2022 Aug 13;4(1):obac020. doi: 10.1093/iob/obac020 (PMC9375770; doi:10.1093/iob/obac020)
Supplement: obac020_Supplemental_File [file obac020_supplemental_file.pdf]

Supplemental Materials

Table S1. Table designating the microhabitat, feeding mode, and literature source for the feeding mode category for each species in the dataset. Species were classified as elastic if they possessed morphological indicators of elastic feeding and are in the same genus as others with demonstrated elastic feeding. Information on microhabitat is from Fabre et al. 2020.

Species	Family	Life cycle	Microhabitat	Feeding mode	Source
Ambystoma gracile	Ambystomatidae	bi	SF	muscle	
Ambystoma maculatum	Ambystomatidae	bi	SF	muscle	Deban et al. 2020
Ambystoma tigrinum	Ambystomatidae	bi	SAq	muscle	
Aneides aeneus	Plethodontidae	dd	S	muscle	Scales et al. 2019
Aneides flavipunctatus	Plethodontidae			muscle	Deban et al. 2020
Aneides hardii	Plethodontidae	dd	T	muscle	Scales et al. 2019
Aneides lugubris	Plethodontidae	dd	A	muscle	Scales et al. 2019
Batrachoseps attenuatus	Plethodontidae	dd	T	spring	Deban et al. 2020
Batrachoseps pacificus	Plethodontidae	dd	T	spring	Scales et al. 2019
Batrachuperus pinchonii	Hynobiidae	bi	Aq	suction	Deban et al. 2001
Bolitoglossa adspersa	Plethodontidae	dd	T	spring	Scales et al. 2019
Bolitoglossa altamazonica	Plethodontidae	dd	T	spring	Scales et al. 2019
Bolitoglossa alvaradoi	Plethodontidae	dd	A	spring	Scales et al. 2019
Bolitoglossa dofleini	Plethodontidae			spring	Deban et al. 2007
Bolitoglossa epimela	Plethodontidae	dd	T	spring	Scales et al. 2019
Bolitoglossa franklini	Plethodontidae	dd	A	spring	Deban et al. 2020
Bolitoglossa lignicolor	Plethodontidae	dd	A	spring	Scales et al. 2019
Bolitoglossa mexicana	Plethodontidae	dd	A	spring	Scales et al. 2019
Bolitoglossa platydactyla	Plethodontidae	dd	A	spring	Scales et al. 2019
Bolitoglossa porrasorum	Plethodontidae	dd	A	spring	Scales et al. 2019
Bolitoglossa rostrata	Plethodontidae	dd	T	spring	Scales et al. 2019
Bolitoglossa rufescens	Plethodontidae	dd	A	spring	Scales et al. 2019
Bolitoglossa salvinii	Plethodontidae	dd	A	spring	Scales et al. 2019
Bolitoglossa subpalmata	Plethodontidae	dd	A	spring	Scales et al. 2019
Chioglossa lusitanica	Salamandridae	bi	SAq	spring	Stinson and Deban 2017
Chiropterotriton chiropterus	Plethodontidae	dd	A	spring	Scales et al. 2019
Chiropterotriton chondrostega	Plethodontidae			spring	Deban et al. 2020
Chiropterotriton magnipes	Plethodontidae	dd	CT	spring	Scales et al. 2019
Chiropterotriton multidentatus	Plethodontidae	dd	A	spring	Scales et al. 2019
Chiropterotriton priscus	Plethodontidae	dd	T	spring	Scales et al. 2019
Desmognathus apalachicolae	Plethodontidae	bi	T	muscle	Scales et al. 2019
Desmognathus fuscus	Plethodontidae	bi	SAq	muscle	Scales et al. 2019
Desmognathus marmoratus	Plethodontidae	bi	SAq	muscle	Scales et al. 2019
Desmognathus ochrophaeus	Plethodontidae	bi	T	muscle	Scales et al. 2019
Desmognathus quadramaculatus	Plethodontidae	bi	SAq	muscle	Deban et al. 2020
Ensatina eschscholtzii	Plethodontidae	dd	T	spring	Deban et al. 2020
Eurycea bislineata	Plethodontidae	bi	SAq	spring	Scales et al. 2019
Eurycea cirrigera	Plethodontidae	bi	Aq	spring	Scales et al. 2019
Eurycea guttolineata	Plethodontidae			spring	Deban et al. 2020
Eurycea longicauda	Plethodontidae	bi	T	spring	Scales et al. 2019
Eurycea lucifuga	Plethodontidae	bi	CAq	spring	Scales et al. 2019
Eurycea neotenes	Plethodontidae	pd	Aq	spring	Scales et al. 2019
Eurycea robusta	Plethodontidae	pd	CAq	spring	Scales et al. 2019
Eurycea sosorum	Plethodontidae	pd	Aq	spring	Scales et al. 2019
Eurycea spelaea	Plethodontidae	pd	CAq	spring	Scales et al. 2019
Eurycea waterlooensis	Plethodontidae	pd	Aq	spring	Scales et al. 2019
Gyrinophilus porphyriticus	Plethodontidae	bi	SAq	spring	Deban et al. 2020
Hemidactylium scutatum	Plethodontidae	bi	T	spring	Deban et al. 2020
Hydromantes genei	Plethodontidae	dd	CT	spring	Scales et al. 2019
Hydromantes imperialis	Plethodontidae	dd	CT	spring	Deban et al. 2020
Hydromantes italicus	Plethodontidae	dd	CT	spring	Scales et al. 2019
Hydromantes platycephalus	Plethodontidae	dd	T	spring	Scales et al. 2019
Hynobius nebulosus	Hynobiidae	bi	T	muscle	Deban et al. 2001; 2007
Ichthyosaura alpestris	Salamandridae	bi	SAq	muscle	Heiss et al. 2016
Lissotriton vulgaris	Salamandridae	bi	SAq	muscle	Heiss et al. 2016
Notophthalmus meridionalis	Salamandridae	bi	Aq	muscle	Scales et al. 2019
Notophthalmus viridescens	Salamandridae	bi	SAq	muscle	Deban et al. 2020
Pachytriton brevipes	Salamandridae	bi	Aq	suction	Deban et al. 2001
Paramesotriton hongkongensis	Salamandridae	bi	SAq	muscle	Deban et al. 2001; 2007
Paramesotriton labiatus	Salamandridae	bi	Aq	suction	Stinson and Deban 2017
Plethodon cheoah	Plethodontidae	dd	T	muscle	Scales et al. 2019
Plethodon cinereus	Plethodontidae	dd	T	muscle	Scales et al. 2019
Plethodon elongatus	Plethodontidae	dd	T	muscle	Scales et al. 2019
Plethodon fourchensis	Plethodontidae	dd	T	muscle	Scales et al. 2019
Plethodon glutinosus	Plethodontidae	dd	T	muscle	Deban et al. 2001
Plethodon grobmani	Plethodontidae	dd	T	muscle	Scales et al. 2019
Plethodon metcalfi	Plethodontidae			muscle	Deban et al. 2020
Plethodon serratus	Plethodontidae	dd	T	muscle	Scales et al. 2019
Plethodon vandykei	Plethodontidae	dd	T	muscle	Scales et al. 2019
Plethodon welleri	Plethodontidae	dd	T	muscle	Scales et al. 2019
Plethodon yonahlossee	Plethodontidae	dd	T	muscle	Scales et al. 2019
Proteus anguinus	Proteidae	pd	CAq	suction	Deban et al. 2001
Pseudoeurycea bellii	Plethodontidae	dd	T	spring	Scales et al. 2019
Pseudoeurycea leprosa	Plethodontidae	dd	T	spring	Deban et al. 2020
Pseudoeurycea lineola	Plethodontidae	dd	SF	spring	Scales et al. 2019
Pseudotriton montanus	Plethodontidae	bi	T	spring	Scales et al. 2019
Pseudotriton ruber	Plethodontidae	bi	SAq	spring	Deban et al. 2020
Salamandra algira	Salamandridae	bi	T	muscle	Scales et al. 2019
Salamandra atra	Salamandridae	vi	T	muscle	Scales et al. 2019
Salamandra corsica	Salamandridae	bi	T	muscle	Scales et al. 2019
Salamandra salamandra	Salamandridae	bi	T	muscle	Deban et al. 2020

(continued)

Species	Family	Life cycle	Microhabitat	Feeding mode	Source
Salamandrella keyserlingii	Hynobiidae	bi	T	muscle	Deban et al. 2001
Stereochilus marginatus	Plethodontidae	bi	Aq	spring	Deban et al. 2020
Taricha torosa	Salamandridae	bi	SAq	muscle	Deban et al. 2001; 2007
Thorius macdougalli	Plethodontidae			spring	Deban and Bloom 2018
Thorius minutissimus	Plethodontidae	dd	T	spring	Deban and Bloom 2018
Thorius narisovalis	Plethodontidae	dd	T	spring	Deban and Bloom 2018
Thorius pennatulus	Plethodontidae	dd	T	spring	Deban and Bloom 2018
Thorius pinicola	Plethodontidae	dd	T	spring	Deban and Bloom 2018
Thorius tlaxiacus	Plethodontidae	dd	T	spring	Deban and Bloom 2018
Tylototriton verrucosus	Salamandridae	bi	SAq	muscle	Deban et al. 2001

Table S2. Results from the phylogenetic ANOVA and MANOVA which includes all species in the dataset.

param	stat	F-value	p-value
All	NA	0.55	0.72
area	NA	0.07	1.00
Elevation	max	0.43	1.00
	med	0.19	1.00
	min	0.08	1.00
	sd	0.67	1.00
Max Precip.	max	0.31	1.00
	med	0.08	1.00
	min	0.50	1.00
	sd	0.21	1.00
Max. Temp.	max	1.37	1.00
	med	0.08	1.00
	min	0.00	1.00
	sd	1.19	1.00
Mean Daily Temp. Range	max	0.02	1.00
	med	0.59	1.00
	min	1.07	1.00
	sd	0.93	1.00
Min. Precip.	max	0.74	1.00
	med	2.83	0.64
	min	1.63	1.00
	sd	0.09	1.00
Min. Temp.	max	1.33	1.00
	med	1.14	1.00
	min	0.65	1.00
	sd	0.01	1.00

Table S3. Loadings of ecological traits from the linear discriminant analysis. Higher values represent variables of greater importance in discriminating between species with ballistic and nonballistic tongues.

trait	LD1
bio2	0.000
bio5	0.014
bio6	0.014
bio13	-0.005
bio14	-0.002
alt	0.001
area	0.252

Table S4. Results from the phylogenetic ANOVA and MANOVA which includes all species except Chioglossa lusitanica in the dataset.

param	stat	F-value	p-value
All	NA	0.53	0.74
area	NA	0.03	1.00
Elevation	max	1.09	1.00
	med	0.40	1.00
	min	0.08	1.00
	sd	1.27	1.00
Max Precip.	max	0.59	1.00
	med	0.12	1.00
	min	0.50	1.00
	sd	0.48	1.00
Max. Temp.	max	2.47	0.92
	med	0.13	1.00
	min	0.09	1.00
	sd	1.90	1.00
Mean Daily Temp. Range	max	0.20	1.00
	med	0.62	1.00
	min	1.24	1.00
	sd	1.65	1.00
Min. Precip.	max	0.61	1.00
	med	2.70	0.72
	min	1.77	1.00
	sd	0.08	1.00
Min. Temp.	max	1.92	1.00
	med	0.91	1.00
	min	0.38	1.00
	sd	0.26	1.00

Table S5. Rate results and associated model from the OUwie analysis for each climatic variable. Bolded rows represent the best fit model for each trait across 100 simmaps.

trait	model	mean lnL	Δ AICc	$\sigma_{elastic}$	σ_{muscle}
alt	BM1	-258.137	1.894		1.175
**alt**	**BMS**	**-256.105**	**0.000**	**1.540**	**0.792**
**area**	**BM1**	**219.694**	**0.000**		**0.000**
area	BMS	219.740	2.076	0.000	0.000
**bio13**	**BM1**	**-86.609**	**0.000**		**0.013**
bio13	BMS	-86.589	2.129	0.013	0.012
bio14	BM1	-8.423	6.129		0.002
**bio14**	**BMS**	**-4.274**	**0.000**	**0.001**	**0.002**
**bio2**	**BM1**	**23.534**	**0.000**		**0.001**
bio2	BMS	24.296	0.645	0.001	0.001
**bio5**	**BM1**	**-40.363**	**0.000**		**0.004**
bio5	BMS	-40.258	1.959	0.004	0.004
bio6	BM1	-71.817	1.267		0.009
**bio6**	**BMS**	**-70.099**	**0.000**	**0.006**	**0.011**

Table S6. Averaged results for 100 OUwie runs using data simulated under multi-rate Brownian motion (BMS). All 100 recover BMS as the best-fitting model, indicating statistical power to distinguish between the two evolutionary models.

Model	AICc	Muscular	Elastic
BM1	473.830	0.636	NaN
BMS	466.751	0.335	0.921

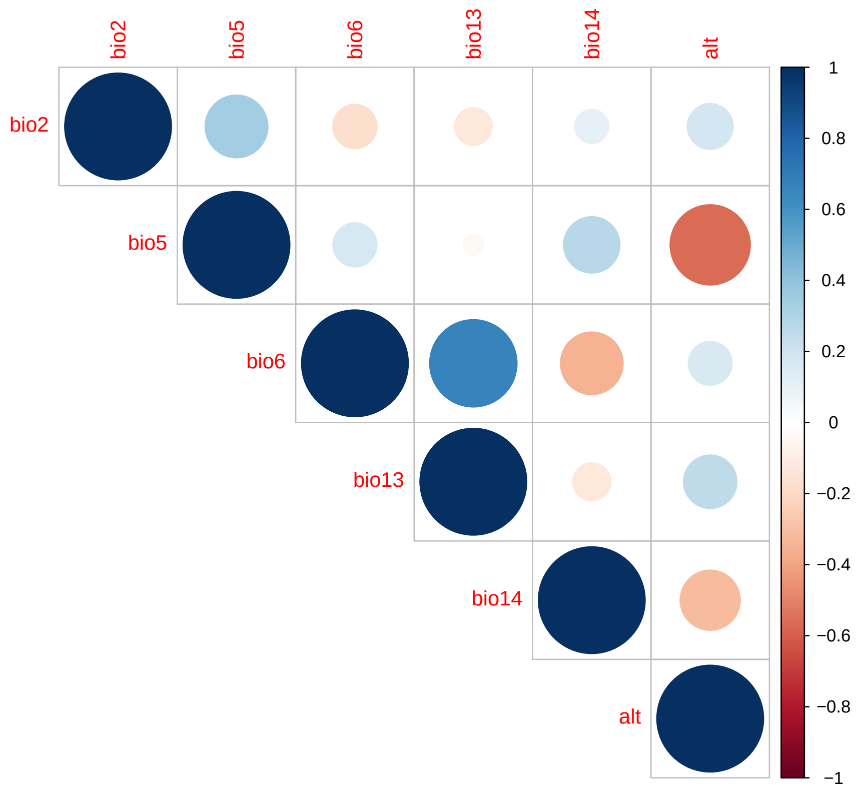

Figure S1. Pearson correlation between each of the remaining bioclimatic variables in the dataset.

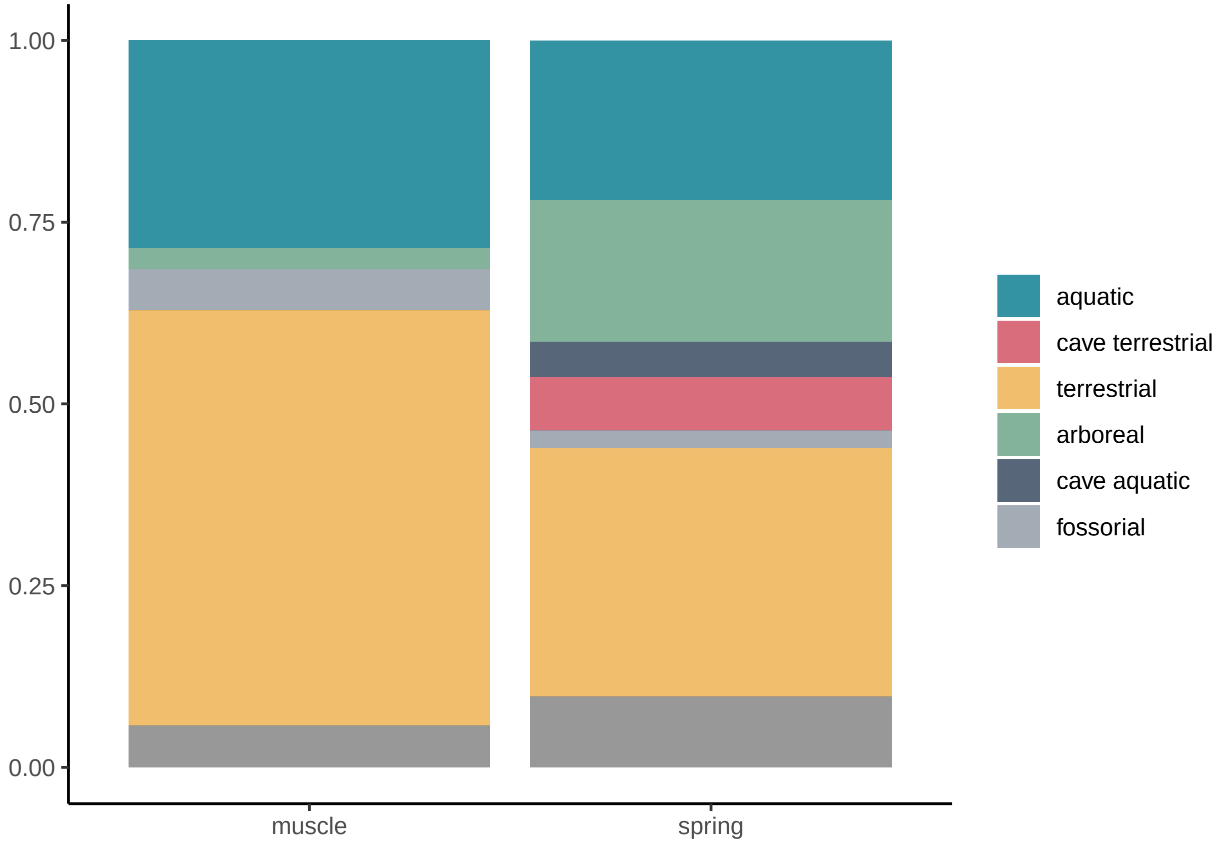

Figure S2. Percentage of species in each microhabitat category by feeding mode.

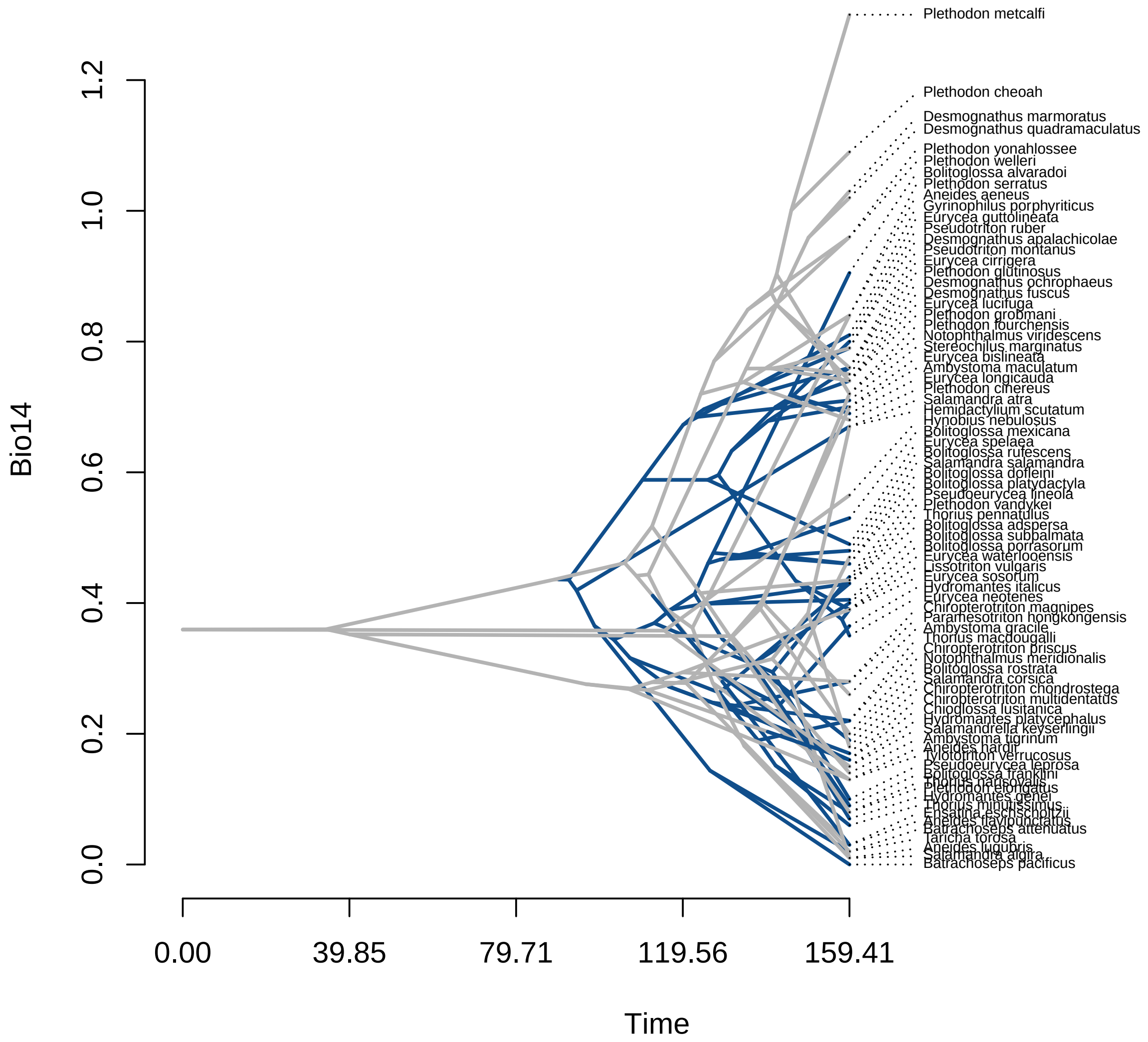

Figure S3. Phenogram of minimum precipitation evolution throughout salamander evolutionary history. Branches on the phylogeny are colored by feeding mechanism (blue: elastic; grey: muscle).
